# To be EndMT or not to be, that is the question in pulmonary hypertension

**DOI:** 10.1007/s13238-015-0183-z

**Published:** 2015-06-23

**Authors:** Jianhua Xiong

**Affiliations:** Center for Molecular Medicine, National Heart, Lung and Blood Institute, National Institutes of Health, Bethesda, MD 20892 USA

Pulmonary hypertension (PH) is a progressive and devastating disease of various causes that is associated with structural and functional disorder and inappropriately increased pressure of pulmonary small- to medium-sized vasculature. Extensive pulmonary vascular remodeling with narrowing lumen is well characterized in all forms of PH, which is hemodynamically defined by a mean pulmonary artery pressure exceeding 25 mmHg at rest (Schermuly et al., 2011; Mehari et al., 2014). The morbidity and mortality of PH continues to increase due to no cure (Mehari et al., 2014); however, our understanding of the mechanism and therapeutics underlying PH remains far from complete. There are many competing hypotheses for how PH develops in a genetic or sporadic way (Schermuly et al., 2011). One of them is that the endothelial-to-mesenchymal transition (EndMT) could be implicated in initiation and progression of human PH (Arciniegas et al., 2007). Notably, this point is further emphasized by two recent papers, which provide direct evidence linking EndMT to PH (Good et al., 2015; Ranchoux et al., 2015).

Vascular remodeling of intimal, medial, and adventitial hypertrophy in PH roughly involves endothelium, smooth muscle, and fibroblasts (Schermuly et al., 2011). Although pulmonary vasculature in PH is thought to undergo a series of structure change events with a complex multifactorial etiology, endothelial cells seem to play a central role in this process in view of the following four major reasons. First, the common plexiform lesions in the vessels of patients with PH generally result from excessive endothelial cell proliferation. Second, endothelial cells act with quite widespread autocrine and paracrine effects via secretion of numerous cell signaling effectors including, but certainly not limited to, nitric oxide, endothelin-1, and serotonin. Third, impaired semipermeable barrier of the pulmonary endothelial lining due to endothelial injuries renders the underlying interstitial cells susceptible to diverse blood-borne factors (Budhiraja et al., 2004). Last, germline loss-of-function bone morphogenic protein receptor type 2 (BMPR2) mutations have been significantly linked to the etiology of familial and idiopathic primary pulmonary hypertension (International et al., 2000; Austin and Loyd, 2014). More importantly, BMPR2 is predominantly expressed in endothelium and tightly controls the permeability of the pulmonary artery endothelial wall (Atkinson et al., 2002; Burton et al., 2011).

EndMT is characterized by the acquisition of mesenchymal- and stem-cell-like properties in endothelium subjected to intrinsic or extrinsic cues and functions as a critical source of fibroblasts in various physiological and pathological settings encompassing heart development, tumor progression, and fibrosis (Lin et al., 2012; Yu et al., 2014). One such notorious fibrosis target organ is lung. Reduced fibrinolytic activity and thereby elevated cellular fibronectin concentration have been demonstrated in the lung vessels of monocrotaline-induced rat PH model (Schultze and Roth, 1993; Schultze et al., 1996). Increased collagen deposition is capable of decreasing the distensibility of hypertensive pulmonary arteries (Tozzi et al., 1994). As mentioned earlier, even dysregulation of the intracellular cytoskeleton network causes altered permeability and morphology of pulmonary endothelium (Dudek and Garcia, 2001). Subsequently, Arciniegas and colleagues observed EndMT in pulmonary artery development of chicken embryos *in vivo* and *in vitro* (Arciniegas et al., 2005). In addition, myocardin promotes the transdifferentiation of pulmonary arteriolar endothelial cells into smooth muscle-like cells in hypoxia-induced rat PH model and porcine pulmonary artery endothelial cells (Zhu et al., 2006). A prior study showed a spontaneous and transforming growth factor β (TGF-β)-induced EndMT in pulmonary endothelial cells isolated from caveolin-1 knockout mice compared to their wild-type littermates (Li et al., 2013). Moreover, the precisely orchestrated EndMT contributes to bleomycin- and radiation-induced pulmonary fibrosis (Hashimoto et al., 2010; Choi et al., 2015). As progressive pulmonary fibrosis can lead to pulmonary hypertension (Rockey et al., 2015), it will be of considerable interest to ask whether EndMT participates in the regulatory network of human PH and, if so, what is the exact role of EndMT in the development of PH?

**Figure 1:**
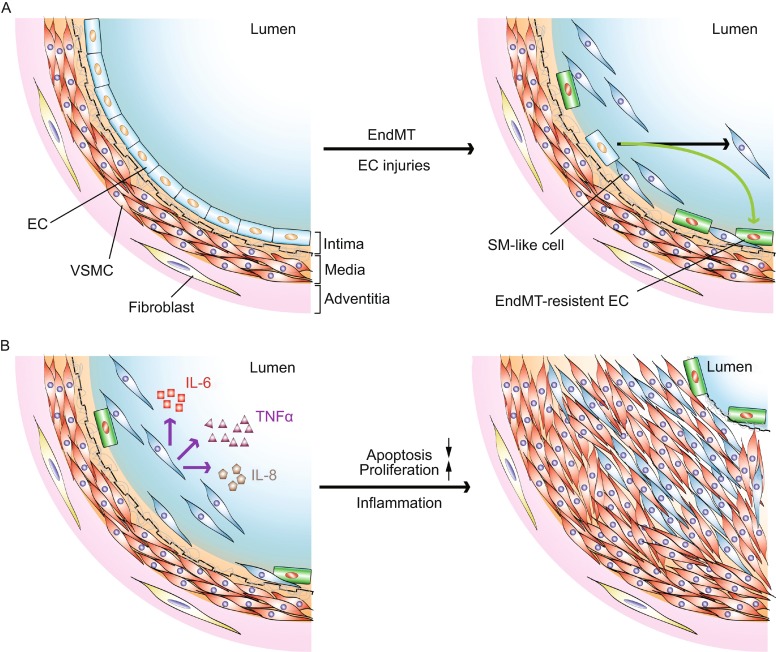
The role of endothelial-to-mesenchymal transition (EndMT) at roughly early (A) and late (B) stages in the pathogenesis of pulmonary hypertension. (A) Genetic and/or microenvironmental insults-induced EC injuries trigger EndMT in pulmonary vasculature. (B) Activated EndMT cells secrete proinflammatory cytokins to potentially stimulate VSMC and/or SM-like cell proliferation and inhibit these cell apoptosis, leading to pulmonary hypertension. Abbreviations: EC, endothelial cell; VSMC, vascular smooth muscle cell; SM-like, smooth muscle-like; IL-6, interleukin-6; IL-8, interleukin-8; TNFα, tumor necrosis factor α

A key step towards answering these questions has been made by two intriguing studies (Good et al., 2015; Ranchoux et al., 2015) (Fig. 1). As some may recall, Qiao and colleagues took advantage of mice that underwent left pneumonectomy and monocrotaline pyrrole injection to establish a mouse model of PH. Endothelial lineage tracing analyses in this PH mouse model reveals that endothelial cells in the pulmonary neointima have detectable smooth muscle gene expression. Likewise, concurrent expression of endothelial cell and smooth muscle markers occurs in human pulmonary arterial hypertension neointimal lesions (Qiao et al., 2014). Consistent with these observations, Good et al. determined the presence of EndMT by assessing the colocalization of von Willebrand factor and α-smooth muscle actin (α-SMA) in the pulmonary endothelium from the hypoxia/SU5416 preclinical murine pulmonary artery hypertension (PAH) model and systemic sclerosis-associated-PAH (SSc-PAH) patients. Furthermore, a panel of functional assays *in vitro* lends further support to the notion that EndMT is a *bona fide* mechanism underlying the pathogenesis of PH (Good et al., 2015). TGF-β has been delineated as a major inducer of EndMT (van Meeteren and ten Dijke, 2012). Nevertheless, utilizing the single reagent TGF-β to galvanize transcription program switching in the EndMT of human pulmonary microvascular endothelial cells seems to require three weeks and several passages (Reynolds et al., 2012). Using a cocktail of TGF-β and inflammatory cytokins tumor necrosis factor α (TNFα) and interleukin 1β (IL-1β) previously described for induction of EndMT in human intestinal microvascular endothelial cells (Rieder et al., 2011), Good et al. obtained the induced EndMT (I-EndMT) cells derived from human pulmonary artery endothelial cells *in vitro* in a more efficient fashion (Good et al., 2015). These I-EndMT cells secrete high levels of proinflammatory cytokins (such as IL-6, IL-8, and TNFα) and exhibit a similar proinflammatory phenotype in patients with human lung fibroblasts SSc-PAH. Next, it was found that the endothelial barrier integrity is significantly compromised in I-EndMT cells, reminiscent of the features in PH endothelium (Budhiraja et al., 2004; Good et al., 2015).

Writing in *Circulation* at nearly the same time, Ranchoux et al. reported that analysis of endothelial cell-cell junction, and endothelial and subendothelial cell phenotype in intimal and plexiform lesions from PAH lungs relative to control non-tumor lung specimens, was carried out using unambiguous endothelial (CD31, CD34, VE-cadherin) and mesenchymal α-SMA markers. Combined with this analysis result, the protein and mRNA expression patterns consolidate the notion of a key role of EndMT in PH pathology (Ranchoux et al., 2015). Additionally, a unique and refined morphological signature in plexogenic pulmonary arteriopathy has been identified thanks to pioneering research efforts (Smith and Heath, 1979; Weibel, 2012). Remarkably, Ranchoux and co-workers applied transmission electron microscopy, and correlative light and electron microscopy, providing unequivocal ultrastructural-level evidence of ongoing dynamic EndMT in PH samples. Next, the EndMT was examined in the context of conventional monocrotaline and SuHx mouse PH models and the novel BMPR2 deficient rat PH model. Indeed, the *in vivo* EndMT characteristics in these mouse models are comparable to those in human PH tissues. Excitingly, partial rescue of EndMT-related gene expression and phenotypes has been achieved by rapamycin (Ranchoux et al., 2015).

The headline finding of these two studies is that the concept of EndMT in PH has been initially proposed on the basis of compelling experimental proofs (Good et al., 2015; Ranchoux et al., 2015). This discovery opens a new therapeutic window for suppressing or even reversing the pathogenic progression in certain common, but genetically defined, subtype of human PH. Each pulmonary endothelial cell in potential or current patients with PH decides whether its next action is to be EndMT or not to be (with apologies to pre-eminent English dramatist William Shakespeare for scrambling the immortal opening phase in his play Hamlet). In the broad landscape, it will be imperative to determine how the EndMT process is orchestrated, and what its context dependencies may be. Future advances in understanding the distinct stage-specific EndMT events and the druggability of EndMT will require a gene expression profile and more functional analyses to unravel the molecular mechanism in a tempo-spatial manner. Finally, these studies offer the possibility of EndMT as a promising pharmaceutical target in human PH, which warrants further investigations.
